# Humanitarian–Development Nexus: strengthening health system preparedness, response and resilience capacities to address COVID-19 in Sudan—case study of repositioning external assistance model and focus

**DOI:** 10.1093/heapol/czad087

**Published:** 2024-01-09

**Authors:** Muna Mohamed Nur, Huzeifa Aweesha, Mahmoud Elsharif, Ahmed Esawi, Arwa Omer, Mohamed Musa

**Affiliations:** Development of Health System Unit, World Health Organisation, Nile Avenue, Othman Digna St., Khartoum 2234, Sudan; Development of Health System Unit, World Health Organisation, Nile Avenue, Othman Digna St., Khartoum 2234, Sudan; Department of Epidemiology and Global Health, Umea University, NUS 5B Destination PA, Umea 90185, Sweden; Development of Health System Unit, World Health Organisation, Nile Avenue, Othman Digna St., Khartoum 2234, Sudan; Institute of Health Policy, Management and Evaluation, University of Toronto, Health Sciences Building, 155 College Street, Suite 425, Toronto M5T 3M6, Canada; International Health Directorate, Federal Ministry of Health, Nile Avenue, Othman Digna St., Khartoum 303, Sudan; University of Medical Sciences and Technology, AlRiyad, Africa Street, Khartoum 12810, Sudan; Development of Health System Unit, World Health Organisation, Nile Avenue, Othman Digna St., Khartoum 2234, Sudan

**Keywords:** Sudan, external assistance, COVID-19, health systems, resilience, HDPNx

## Abstract

The advent of the COVID-19 pandemic and the establishment of a new transitional government in Sudan with rejuvenated relations with the international community paved the way for external assistance to the EU COVID-19 response project, a project with a pioneering design within the region. The project sought to operationalize the humanitarian–development–peace nexus, perceiving the nexus as a continuum rather than sequential due to the protracted nature of emergencies in Sudan and their multiplicity and contextual complexity. It went further into enhancing peace through engaging with conflict and post-conflict-affected states and communities and empowering local actors. Learning from this experience, external assistance models to low- or middle-income countries (LMICs) should apply principles of flexibility and adaptability, while maintaining trust through transparency in exchange, to ensure sustainable and responsive action to domestic needs within changing contexts. Careful selection and diverse project team skills, early and continuous engagement with stakeholders, and robust planning, monitoring and evaluation processes were the project highlights. Yet, the challenges of political turmoil, changing Ministry of Health leadership, competing priorities and inactive coordination mechanisms had to be dealt with. While applying such an approach of a health system lens to health emergencies in LMICs is thought to be a success factor in this case, more robust technical guidance to the nexus implementation is crucial and can be best attained through encouraging further case reports analysing context-specific practices.

Key messages

- External assistance can be utilized to implement a humanitarian–development nexus (HDPNx) approach in complex settings while contextualizing the approach and responding to domestic needs during emergencies and pandemics. Donors are encouraged to provide external assistance with principles of flexibility and adaptability.
- Advocacy is needed for the HDPNx approach and fostering funds that allow for it, as is developing operational guidelines and experience sharing that address low- or middle-income countries’ contexts and needs.
- Multi-disciplinary teams and prolonged engagement and discussion with all stakeholders are crucial to improve the development and implementation of nexus projects, with recorded successes.
- Responding to health emergencies using a health system lens is a clear example of the HDPNx approach.

## Introduction

Since the beginning of the COVID-19 pandemic, Sudan’s health system has faced multiple shocks and disruptive events, underscoring the need to build the health system’s resilience (HealthCluster, 2020). The prevailing fragile health system situation was further exacerbated by the unprecedented political turmoil and instability following the December 2018 revolution and continuing conflicts (WHO, 2022). Within this volatile post-revolution scene more global support for Sudan was seen in the establishment of multiple ‘Friends of Sudan’ initiatives (Anon, 2019). COVID-19 has triggered huge external funds to Sudan’s health sector, with a dominating nexus approach that relies on a health system lens to address protracted and new health emergencies and address essential health needs (WHO, 2020). Windows of opportunity were opened to adopt new ways of working in this extraordinary global and local context.

This practice report describes the experience of utilizing external assistance to strengthen health system resilience to enable the system to better prevent, detect and respond to long-standing health emergencies by operationalizing the humanitarian–development nexus (HDPNx) approach within the context of Sudan. HDPNx for health here is defined as ‘any health-related activity where at least two of the three groups of actors (humanitarian, development and peace) work together with the aim of providing immediate life-saving and life-supporting assistance; strengthening or rebuilding national systems, institutions and capacities; strengthening emergency management capacities; or addressing emergencies drivers (WHO, 2021a). In this paper, we discuss the implementation successes, enablers and limitations of adopting this external assistance nexus model and how they were handled, and offer our recommendations on the best ways to address them in the future to meet the challenges of COVID-19 and future pandemics. Hence, it is hoped that this report will inform project designs and decisions that are better suited for low- or middle-income countries (LMICs) with complex contexts and also assist required reforms of external assistance design and the application of such models with greater adaptability and responsiveness to comparable domestic needs and contexts.

The report applied the conceptual framework of the health systems for health security (WHO, 2021b) with some adaptations. The data sources used include project progress reports and reviews, focus group discussions and interviews with key stakeholders. The analysis adopted a qualitative design which reflected our experience as implementers of this external assistance within the nexus approach. This includes desk review of all documents and data compilation, followed by thematic analysis (Dahlgren *et al.*, 2007) when following the framework through team deliberations, highlighting the successes, challenges and lessons learned. The EU COVID-19 Sudan Response Project is funded by the European Union Emergency Trust Fund for Africa and implemented through WHO with an objective of achieving sustainable strengthening of the health system for epidemic preparedness and response addressing the needs of the COVID-19 pandemic in Sudan. In consultation with the Ministries of Health (MOHs), 10 of Sudan’s 18 states were chosen as the most vulnerable and in need. The project was co-managed by the Emergencies and Health Systems Unit in the country office, which provided the nexus for implementation interventions, and state officers in many selected states. The 36 month, 20-million-euro project has been extended. Even though COVID-19 is no longer a public health emergency of worldwide concern, the nexus strategy and breadth allowed us to continue health system strengthening to prepare for future public health emergencies.

## Implementation

In 2019, the EU COVID-19 Sudan Response Project was initiated with the objective of improving the health system’s preparedness and response to outbreaks and other health emergencies, specifically the COVID-19 pandemic. Through this project, the EU as a financier and WHO as an implementing agency also aimed to address the major issue of sustainability. Many external actors supported service delivery without considering sustainability. This often resulted in parallel systems that disengaged MOHs and other domestic players, creating challenges in ensuring that services continued to beneficiaries after external assistance ended.

The EU has a long history of undertaking initiatives in conjunction with partners that adopt the HDPNx approach and supports the implementation of sustainable interventions in Sudan. After signing the Qatar peace agreement in 2011, a dialogue was initiated to transform from humanitarian to developmental support. Later between 2015–2016 the HDPNx approach started to emerge, and the EU played a major role advocating for this concept. The EU has been working with the WHO on health system strengthening projects in Sudan for many years which provided valuable insights and good examples on how to sustain humanitarian interventions through the lens of health system strengthening and what can be done to support durable interventions that can respond to recurring emergencies in Sudan. This history and experience in implementing HDPNx initiatives in Sudan, as well as the trust developed between donor and implementer helped in preparing the road for the EU COVID-19 Response Sudan Project when COVID-19 struck.

The project was designed with a holistic approach, recognizing the integral and inseparable relationship between the health system and emergency response functions. The project’s design was based on several principles, including a comprehensive understanding of the functions of the health system, the integration and alignment of humanitarian and development efforts, and flexibility and responsiveness recognizing that the contexts of Sudan and the pandemic are constantly changing and interventions must adapt accordingly.

The project began by assembling a team of experts with diverse skill sets and areas of expertise. The team selection process was crucial to the success of the project and took a significant amount of time to ensure the right people were in the right place. During the inception phase of the project implementation, the project team ensured early engagement with MOHs both at federal and state levels and together conducted a comprehensive baseline survey followed by joint development of a micro-plan. This was crucial to enhance ownership, respond to priority needs and align with ongoing national reforms. Results of the baseline survey and identification of gaps revealed the urgency for establishment of sub-national permanent isolation facilities and the strengthening of the health system’s capacity to respond to health emergencies. Five main outcomes were formulated: strengthening governance and coordination, strengthening surveillance, rapid response and lab capacities, strengthening capacities to isolate and mange cases, strengthening risk communication and water, sanitation and hygiene (WASH) measures at facility levels, and maintaining essential primary and emergency care services. The project initially targeted 6 states out of a total of 18 but later more funds were mobilized to support 4 more states. The 10 states were selected according to stringent criteria and a needs assessment and focusing on the most vulnerable and conflict-affected states.

The project also sought to contribute to learning about operationalization of the HDPNx approach through continuous dialogues within the team, with the donor and with key stakeholders both at local and regional level. As this is a relatively new concept and there is a lack of practical guidance in its operationalization, many ideas emerged through these discussions. In our experience, the nexus was perceived and implemented as a continuum rather than sequential due the protracted nature of emergencies in Sudan and their multiplicity and complexity in a changing context.

A robust monitoring and evaluation (M&E) framework was developed, with periodic meetings and reporting with the project team and stakeholders that provided an opportunity for the team to share updates on progress, discuss challenges and make adjustments. Monthly donor check-ins also provided an opportunity to provide feedback on the project and facilitated budget reallocation and response to evolving situations. An online system was also implemented to track progress, providing real-time implementation data. Progress reports were disseminated periodically with stakeholders demonstrating transparency and building the needed trust between donors, implementers and beneficiaries.

During the initial phases of developing the project design, a national task force was formulated and charged with steering the project implementation, providing inputs on the development of activities and ensuring delivery of the project outputs. The task force was headed by Federal Ministry of Health (FMOH) and comprised of members from the different departments. The task force guided an initial risk and vulnerability assessment as part of the baseline survey, which identified at-risk states for COVID-19 and other outbreaks using different tools, and benchmarked the project indicators. Unfortunately, due to constraints imposed by the pandemic, competing priorities and high turn-over in FMOH, the momentum of the national task force was not sustained.

We ensured accountability was in place through transparent communication with beneficiaries, alignment with standards, and by being responsive through frequent briefing with officials and participating stakeholders. Several community consultations with key leaders were conducted to reach consensus on allocating land needed to build the new public health laboratory and isolation centres. These consultations engaged communities on decisions related to their health and responded to their concerns and queries. Joint monitoring visits with the donor were implemented to several states which enhanced trust building, allowed donors to hear directly from beneficiaries and amplified on-the-ground understanding of contextual factors that enhance or impede project implementation. These also provided opportunities to sustain funds for future projects and meet donor expectations.

The project interventions enhanced peacebuilding through interventions targeting conflict and post-conflict affected states and through community engagement activities focusing on strengthening community-based surveillance and providing extensive support to community rapid response teams. Blue Nile state is a poor and underdeveloped state that has suffered from long years of conflict and neglect. A peace agreement brought some hope to communities who have poor access to health and other essential services. Empowering communities in this state and their engagement in health interventions, including emergency preparedness and response, is a strong peacebuilding tool. We targeted newly accessible areas in Blue Nile state (Ulu and Bau) through a WHO/MOH joint training on community-based surveillance that aimed to strengthen surveillance, reporting, verification and response systems for early detection of public health threats and emergencies. These areas were inaccessible to humanitarian aid for over two decades. This training targeted community volunteers and leaders, as the scarcity of a qualified and trained health cadre in this state and difficulties in reaching remote areas makes the involvement of local communities a suitable option, as shifting tasks to them enables sustainability of support.

## Achievements/enablers

The project had special focus on local governance and strengthening community health systems through supporting the formation of needed structures, developing plans, improving capacities and enhancing community engagement. In the context of Sudan, local health systems can play a major role in emergencies preparedness and response. Local health system-strengthening working groups, that included all stakeholders and were routinely attended by state governors and high-level officials, were established. These groups were formulated by decrees from higher levels of government; and we offered support for conducting meetings and evaluation of local health systems, joint supervision missions and advocacy work. Major successes were recorded, for instance, in Red Sea state through this platform >600 health cadres (mainly midwives and medical assistants) were deployed by local resources to remote areas with inadequate service coverage. We also supported community rapid response teams, capacity building and the strengthening of community-based surveillance.

Responding to the insufficient capacities, weak preparedness and infrastructure, the project allocated funds for construction and rehabilitation which included the construction of new sub-national isolation centres and laboratories, and rehabilitation works. The project since its initiation has adopted an all-hazards approach, foreseeing that these isolation centres will respond to all emergencies as there are common elements (and capacities required) in the management of different types of risks.

To achieve more synergistic working relationships between health security, health systems and other sectors for multisectoral and multidisciplinary management of health emergencies, the project collaborated with a variety of actors and stakeholders including other governmental ministries and agencies, UN agencies and non-governmental partners. This was notably true in areas of strengthening governance and coordination, strategic and operational planning both at national and sub-national level, capacity building, and information sharing, monitoring and evaluation. Reviewing the National Public Health Law and Quarantine Law was a great achievement as these laws needed to be updated with current global and local changes, realizing that they did not allow for optimum response to emergencies when COVID-19 hit, and to create an enabling legal environment.

Partners’ coordination mechanisms were activated at federal and state levels, and local health system resilience-building training was synergistically developed and implemented with other partners (e.g. GOAL), contextualizing a module from the Royal Tropical Institute-Amsterdam, which was adopted by the MOH.

More than 3800 health personnel were trained in areas of local health system, planning and M&E, surveillance, case management, infection, prevention and control, laboratory diagnosis, supply chain management etc.

An effective strategy to implement the project successfully was full engagement of beneficiaries, with flexible planning and adjustment of priorities and needs with the evolving pandemic situation and the dynamic nature of the emergency-prone context.

An enabler was the multidisciplinary team that came from diverse backgrounds and specialities who provided the expertise needed to implement the wide scope of the project and discuss challenges and propose solutions from different perspectives, allowing for efficient and effective implementation modalities. Sub-national physical presence was essential to ensure close collaboration with state authorities, response to urgent needs and overall field project oversight.

Through all of this the project built on conceiving the HDPNx as a continuum rather than moving one way or the other. The approach in our project aimed at embedding resilience and sustainability in its interventions rather than making a clear distinction between humanitarian life-saving interventions and long-term developmental ones. We believe both can continue simultaneously to build systems, especially in our emergency protracted context where moving from one intervention to the other may not be feasible.

## Challenges and constraints

The ongoing political turmoil in Sudan following the revolution and later 2021 coup, with poor governance of external assistance, posed considerable challenges in aligning donor funds, often with donors tending to create parallel systems and causing difficulties in coordination and collaboration between different partners. Previous trials of shifting the mindset of partners and policymakers away from the classical humanitarian approach, as well as the tendency to advocate for moving from humanitarian work to development, created wrong assumptions and fears. The concept of HDPNx is poorly understood by many stakeholders and requires vigorous lobbying.

Following the military coup, funding was at risk of being frozen due to the instability of higher-level governmental structures and leadership and having to deal with a de facto government. UN agencies and other donor’s produced strict policies that compromised communication with the MOH and led to the introduction of new and restrictive financing procedures. The post-coup arrangements shifted the mandate to focus solely on humanitarian aid, causing implementation constraints and delays.

The MOH directorates faced difficulties in agreeing on priorities, adding to the disordered response at the start of the COVID-19 pandemic. Multiple activities needed to be implemented concurrently, putting pressure on staff capacity. Great differences in epidemiological COVID-19 and other emergency situations and needs in the different states were recorded, hence we had to deal with different consultations per state. High turnover of MOH staff was a big concern especially at the senior level, which we tried to mitigate by putting more effort into advocacy for the project at all levels.

Coordination of the project activities with other external actors and creating linkages with other COVID-19 projects was a notable challenge due to the absence of an institutional or organizational culture that elevates the importance of integration and emphasizes the added value and sense of coordination with other partners, the competitive culture and behaviour between partners, conflicting interests and the prioritization of one’s own achievement over common goals. Poor communication, information sharing and lack of a common communication language hinders integration. Donor-driven verticality also hampered the ability to join forces in different activities. Accordingly, the team followed a bilateral coordination modality with other partners, for e.g. with Global Fund through the COVID-19 platform to ensure complementarity and avoid duplication. There was some coordination of partners like United Nations Children’s Fund and United Nations Development Programme in areas of Risk Communication and Community Engagement and WASH.

Implementing the approach itself was a significant challenge as actors have fundamentally different principles and approaches for action (e.g. humanitarian action versus development cooperation). These different actors can have incongruent planning cycles with different implementation timelines, which results in scattered planning and precludes having joint monitoring and evaluation frameworks. Even when joint priorities exist and plans align, they can be disrupted by the occurrence of emergencies and other hazardous situations.

## Conclusion

The EU COVID-19 response project is a one-of-a-kind initiative that utilized external assistance to respond to domestic needs during a crisis. The project adopted the HPDNx approach, contextualizing it to a challenging and volatile situation where humanitarian and development interventions were implemented concurrently. Our experience demonstrates that external assistance works in LMICs experiencing political unrest only if actors are invested in fostering trust and applying the principles of flexibility and adaptability to achieve successful outcomes.

## Supplementary Material

czad087_Supp

## Data Availability

The data underlying this article are available in the article and in its online supplementary material.

## Reflexivity statement

The authors include two females and four males with varying levels of seniority. Two of the authors are PhD students. All authors have master’s degrees in Pubic health, health economics or international health with extensive experience in health systems strengthening. Two authors are dentists, three are medical doctors and one is a pharmacist. One author is lead health economist with a senior leadership position in health financing. All authors have good experience in manging health projects and reforms in Sudan.

## Disclaimer

In mid-April 2023, after a lengthy period of political tension, armed conflict erupted between Sudan Armed Forces (SAF) and the Rapid Support Forces (RSF), initially in Khartoum and then widely spreading to other states causing the most devastating political, economic and social turmoil in the recent history of Sudan. We are still unable to predict the impact of conflict of project implementation as the situation is rapidly evolving, but the project has come to a partial halt.

The views expressed herein can in no way be taken to reflect the official opinion of the European Union.

## References

[R1] Anon . 2019. Friends of Sudan supports the planned reforms of Sudan’s economy. Ventures Africa.

[R2] Dahlgren L, Emmelin M, Winkvist A. 2007. Qualitative methodology for international public health. In: *Epidemiology of Public Health and Clinical Medicine*. 2nd edn. Umea University.

[R3] HealthCluster . 2020. Responding to Multiple Emergencies in Sudan. World Health Organization. https://healthcluster.who.int/newsroom/news/item/07-10-2020-responding-to-multiple-emergencies-in-sudan, accessed 19 January 2022.

[R4] WHO . 2020. Humanitarian-Development-Peace Nexus for Health Profile: Sudan. World Health Organization Regional Office for the Eastern Mediterranean. https://apps.who.int/iris/bitstream/handle/10665/351258/9789290227816-eng.pdf?sequence=1&isAllowed=y, accessed 17 June 2022.

[R5] WHO . 2021a. Bridging the Divide: A Guide to Implementing the Humanitarian-Development-Peace Nexus for Health. World Health Organization Regional Office for the Eastern Mediterranean. https://apps.who.int/iris/handle/10665/351260, accessed 17 June 2022.

[R6] WHO . 2021b. Health Systems for Health Security: A Framework for Developing Capacities for International Health Regulations, and Components in Health Systems and Other Sectors that Work in Synergy to Meet the Demands Imposed by Health Emergencies. World Health Organization. https://www.who.int/publications/i/item/9789240029682, accessed 17 June 2022.

[R7] WHO . 2022. Country Cooperation Strategy for WHO and Sudan 2022–2025. WHO Regional Office for the Eastern Mediterranean. https://apps.who.int/iris/bitstream/handle/10665/353562/9789290229698-eng.pdf?sequence=1#:∼:text=The%20Country%20Cooperation%20Strategy%20for,over%20the%20next%20four%20year, accessed 17 June 2022.

