# Supplementary material for: Humanitarian–Development Nexus: strengthening health system preparedness, response and resilience capacities to address COVID-19 in Sudan—case study of repositioning external assistance model and focus
Source: Health Policy Plan. 2024 Jan 9;39(3):327–31. doi: 10.1093/heapol/czad087 (PMC10929768; doi:10.1093/heapol/czad087)
Supplement: czad087_Supp [file czad087_supp.zip › Key political events impact on Nexus implmentation.pdf]

IMPACT OF KEY POLITICAL EVENTS ON NEXUS IMPLEMENTATION

Before 2018

Continuous armed conflicts/ Huamntarian support mainly

Sudan’s underdevelopment status caused by several years of armed conflict coupled with complete cease of development efforts reinforced the fragility of the health system and resulted in Sudan’s dependence on foreign aid in financing its public health programs

2019

Sudan Revolution

The unprecedented political change have attracted more donors and international community to support the new transition. An e.g. "Friends of Sudan" Initiative and peace agreement

2020

COVID-19

New threats to the fragile health system which attracted more partners to support Sudan adopting new approaches such as nexus approach

2021

Military Coup

In OCT 2021 the military took over the power from the transitional government, this resulted in many critical donors refraining from direct collaboration with government and the pause of many developmental projects

2023

The Conflict between SAF and RSF

In April 2023 an armed conflict erupted between SAF and RSF, the impact of this is total suspension of many projects supported by donors and reprograming of support toward humanitarian approach .
