# Supplementary figures and images for: Humanitarian–Development Nexus: strengthening health system preparedness, response and resilience capacities to address COVID-19 in Sudan—case study of repositioning external assistance model and focus
Source: Health Policy Plan. 2024 Jan 9;39(3):327–31. doi: 10.1093/heapol/czad087 (PMC10929768; doi:10.1093/heapol/czad087)

## Slide 1
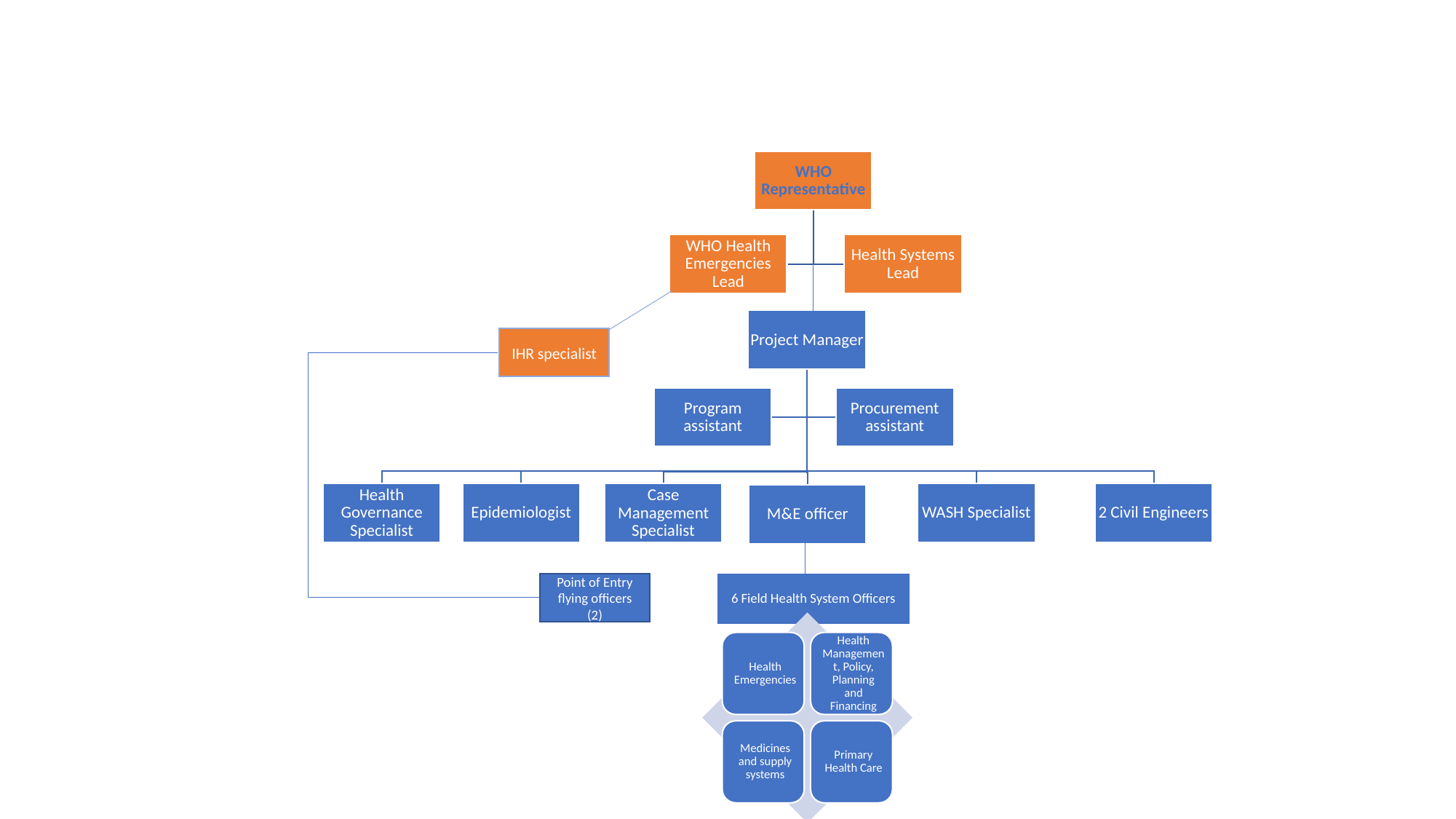

IHR specialist
Point of Entry flying officers (2)

Supplement: czad087_Supp [file czad087_supp.zip › Project organogram.pptx]
