# Supplementary material for: Humanitarian–Development Nexus: strengthening health system preparedness, response and resilience capacities to address COVID-19 in Sudan—case study of repositioning external assistance model and focus
Source: Health Policy Plan. 2024 Jan 9;39(3):327–31. doi: 10.1093/heapol/czad087 (PMC10929768; doi:10.1093/heapol/czad087)
Supplement: czad087_Supp [file czad087_supp.zip › HPPJan 0045r.1 Response to Reviewers.docx]

Tracking of Revisions:
1. Responding to the reviewers’ comments and to provide the requested background, details and more clarity on project activities was difficult to comply with without exceeding the limited word count number. One reviewer has suggested that additions can appear in text boxes outside the main text.
2. Even though a lot of details were provided on the project, but we feel this is important to reflect on the magnitude of work and efforts being done from a diverse team with different backgrounds and specialties.

Responding to the first reviewer:
1. Responding to the first and second reviewer ,a definition of HDPNx was added to explain the theoretical concept and advantage it will bring to health system performance with a reference added.
2. Reference added to WHO guide to HDPNx for health.
3. A diagram added on project team structure which is an important enabler to implement the nexus approach.
4. A paragraph was added to give more details on project( funding, duration, targeted states and management). Links have been provided to publicly available documents and reports should the reader be interested on more details on project design and outcomes. We feel it was worth noting that nexus scope allowed us to continue implementation despite declaration of ‘the end’ of Covid-19.
5. A diagram of timeline of key events have been added that reflect important political events that add to complexity of health emergency context and its impact on external assistance and channeling of funds between humanitarian and developmental needs.
6. One paragraph added to reflect how some project interventions were used as peace-building tools in conflict and post-conflict settings.
7. Links for more details on project:
https://trust-fund-for-africa.europa.eu/where-we-work/regions-countries/horn-africa/sudan_en
Action document:
https://trust-fund-for-africa.europa.eu/our-programmes/humanitarian-development-nexus-strengthening-preparedness-andresponse-health-system-addressing-covid_en
Monitoring and evaluation reports
https://trust-fund-for-africa.europa.eu/results/monitoring-and-evaluation_en
8. References revised.
9. A disclaimer added on impact of recent conflicts on project implementation/ plus a statement that the manuscript does not reflect opinions of the EU( to comply with agreement with EU)


Responding to the second reviewer:
1. Abstract edited to summarize key findings of the intervention.
2. We have added a paragraph on difficulties on engaging with external actors who were involved in Covid-19 response and reasons behind those challenges. This was added on the challenges section. There were no specific TOR/MOU developed with external actors.
3. Community engagement interventions have been through support to strengthening community based surveillance and extensive support to community rapid response teams , this is added as last paragraph to implementation section and linked to interventions that support peace-building.
4. To respond to accountability and governance systems, we added a paragraph on implementation section describing national task force and accountability mechanisms including joined monitoring visits and community consultations. The community consultations were an important tool to agree with communities residing near to construction sites and engage them prior to implementation ensuring they know importance of these facilities and how they will serve these communities and to respond to their concerns and queries.
5. Effective strategies as well as enablers were added.
6. Conclusion amended to reflect that nexus can be implemented if actors invested in fostering trust and applying principles of flexibility and adaptability.
